# Honey dressing for burns

**DOI:** 10.4103/0970-0358.73478

**Published:** 2010

**Authors:** M. Subrahmanyam

**Affiliations:** Department of Surgery, Bharati Vidyapeeth University Medical College and Hospital, Sangli, Maharashtra, India

Sir,

With reference to the article on honey treatment for burns by Baghel *et al*.[1] and comments by Wiwanitket,[2] the following points may be noted. Jull *et al*.[3] in their intervention review had stated “recently trials have evaluated the effects of using honey to help wound healing in both acute wounds (for example, burns, lacerations) and chronic wounds (for example, venous leg ulcers, pressure ulcers). Although honey may improve healing times in mild to moderate superficial and partial thickness burns compared with some conventional dressings, it was found that honey dressings used alongside compression therapy do not significantly increase leg ulcer healing at 12 weeks. There is insufficient evidence to guide clinical practice for other wound types.” Thus, the interpretation of Wiwanitket[2] about the review conclusions does not reflect what the authors stated. In partial thickness and superficial burns, the review repeatedly mentioned that honey may help the wound healing, though with caution, since all the studies were conducted at the same centre and need replication. Studies like the present one are a step in that direction.

Honey may contain botulinum, and hence it is necessary to sterilize honey by gamma irradiation, but majority of the clinical studies used raw honey and no adverse effects have been reported so far.[4]
